# Exploration of combined physical activity and music for patients with Alzheimer’s disease: A systematic review

**DOI:** 10.3389/fnagi.2022.962475

**Published:** 2022-08-08

**Authors:** Kailimi Li, CanCan Cui, Haipeng Zhang, Luning Jia, Rui Li, Hao-Yu Hu

**Affiliations:** ^1^School of Exercise and Health, Shanghai University of Sport, Shanghai, China; ^2^Department of Sport Rehabilitation, Shanghai University of Sport, Shanghai, China; ^3^College of Music and Dance, Guangzhou University, Guangzhou City, China; ^4^Department of Sport Rehabilitation Medicine, Shanghai Shangti Orthopedic Hospital, Shanghai, China

**Keywords:** physical activity, music therapy, Alzheimer’s disease, rehabilitation, non-pharmacological

## Abstract

**Objective:**

Alzheimer’s disease (AD) can be treated in different ways, one of which is combined physical activity and music intervention, which is a non-pharmacological one. This study provided a thorough systematic review on the application of combined physical activity and music intervention in patients with AD.

**Method:**

Online sources, such as PubMed, Web of Science, SAGE Premier, EBSCO, and Cochrane, published from January 2002 to March 2022 were searched for articles. Reviewer screened articles on inclusion criteria and identified relevant studies. 200 studies were selected as potentially relevant; of these, eight met all the inclusion criteria.

**Results:**

The systemic review looked at eight studies, two of which had high methodological quality and six were of moderate quality. Various types of research were included: randomized controlled tails, single-subject study, crossover study, and case report. Music intervention was conducted during an exercise program in six studies. A cognitive stimulation was applied with music therapy and physical activities among two studies. The physical activities or movements included balance training, stretching, strengthening, and different sports activities. Outcome assessment, including the Barthel index in two studies and the functional independence measure, was conducted to evaluate the daily functional score. Mental health was evaluated by Mini-Mental State Examination in three studies.

**Conclusion:**

Combined physical activity and music intervention are beneficial and improve the cognition, function and well-being of patients with AD. Supporting combined physical and music intervention will play a key role in helping clinical guidelines for both physical therapists and music therapists.

## Introduction

Alzheimer’s disease (AD) is the major cause of dementia worldwide, partly because its prevalence continues to grow as the world’s population ages (Qiu et al., 2009; Arvanitakis et al., 2019; Soria Lopez et al., 2019). AD is a common neurological disease that begins with memory loss and gradual loss of abilities to call and respond to the surroundings (CDC, 2020). In addition, according to the report of Centers for Disease Control and Prevention, there are more than 5.8 million Americans living with AD at the end of 2020 (CDC, 2020). The annual cost per patient was approximately 19,144 dollars in 2015, and it is expected to be 507.49 billion in 2040 (Jia et al., 2018). Furthermore, currently, pharmacotherapies seem to slow down the progression of AD. However, the normal pharmacological treatments only target the symptoms of AD, but cannot pause or modify the progression of AD (Tang and Taghibiglou, 2017). Moreover, there are still some limitations, such as limited efficacy and substantial side effects (Jiang et al., 2012). Due to the current prevalence of AD, the situation highlights the urgent need of applying effective non-pharmacotherapies to treat AD patients.

Combined physical activity and music intervention is a non-pharmacological therapy that combines music and physical exercises during the treatment process (Wittwer et al., 2013; Anderiesen et al., 2014; Satoh et al., 2014; Brett et al., 2016; van der Wardt et al., 2017). The phrase “physical activity” refers to any skeletal muscular movement that causes energy expenditure (Sharif et al., 2018). These movements include sports, structured exercises, or dance lessons (Terry et al., 2020). Physical activities have various advantages for elderly adults with regards to their mental and physical health, and music interventions or music based interventions have been noted to improve various types of physical activities (Terry et al., 2020). In addition, four main music-based interventions are commonly distinguished in therapeutic practice, which may overlap or be mixed, namely, music-based activities, such as choir singing or drumming, that include improvising, listening, recreating, and composing (Stegemann et al., 2019). Previous studies have proved that music therapy can be applied to the clinical practice of various neurological and psychiatric disorders (Boso et al., 2006; Baker et al., 2021). Furthermore, combined physical activity and music interventions produce considerable benefits for patients with AD, including improved heart rates, skin conductance, motor patterns, neuroendocrine response, and immunological functions (Ooishi et al., 2017). With combined physical activity and music intervention, positive effects have been explored on psychomotor speed and memory on AD patients (Wittwer et al., 2013; Anderiesen et al., 2014; Brett et al., 2016; Satoh et al., 2017; van der Wardt et al., 2017). More recent research has verified that while implementing physical exercise and music intervention simultaneously, physical exercise has displayed positive effects on AD patients, but no positive effects have been discovered from music intervention alone (Pitkänen et al., 2019). On this basis, the employment of combined physical activity and music intervention on patients with AD and its effect has not reached a consensus. With its universal appeal, combined physical activity and music intervention can be an innovative adjunct to standard pharmacologic therapy for patients with AD.

This systemic review is to determine whether combined physical activity and music intervention is effective for AD patients. We focus on recent systematic reviews in the literature to gain a better understanding of evidence-based best practices in the promotion of combined physical activity and music intervention for health benefits among patients with AD based on studies worldwide. We hypothesize that a combined physical activity and music intervention, as suggested by numerous international health and fitness organizations, will be associated with significantly and clinically relevant health benefits, particularly in patients with AD. We hope that our study will provide a more comprehensive understanding of combined physical activity and music intervention for patients with AD. This study will provide a more comprehensive understanding of combined physical activity and music interventions for patients with AD.

## Methods

### Search strategy

The online databases, namely, PubMed, Web of Science, SAGE Premier, EBSCO, and Cochrane, were applied to identify publications in the field. We edited strategies of these databases into the following table (Table 1).

**TABLE 1:** Search strategy table.

Search strategy for PubMed, Web of Science, SAGE Premier, EBSCO, and Cochrane.	
**Using PubMed database**	
1	TI/AB = “dementia” OR “cognitive impairment*” OR “cognitive dysfunctions” OR “neurocognitive disorder, mild” OR “cognitive decline” OR “mental deterioration”
2	TI = “exercise” OR “train” OR “training” OR “physical activity” OR “physical activities” OR “strength” OR “endurance” OR “resistance” OR “stability” OR “walk*” OR “tai chi” OR “yoga” OR “motor control” OR “core control” “stretch*” OR “run” OR “muscle energy technique” OR “pilates*” OR “hydrotherapy” “water sports” OR “kinesitherapy”
3	TI/AB = “Music” OR “music therapy” OR “sing” OR “rhythm*” OR “tempo*” OR “Music Therapy”
4	Strategy 1 AND 2 AND 3
**Using Web of Science database**	
1	TS = “dementia” OR “cognitive impairment*” OR “cognitive dysfunctions” OR “neurocognitive disorder, mild” OR “cognitive decline” OR “mental deterioration”
2	TI = “exercise” OR “train” OR “training” OR “physical activity” OR “physical activities” OR “strength” OR “endurance” OR “resistance” OR “stability” OR “walk*” OR “tai chi” OR “yoga” OR “motor control” OR “core control” “stretch*” OR “run” OR “muscle energy technique” OR “pilates*” OR “hydrotherapy” “water sports” OR “kinesitherapy”
3	TS = “Music” OR “music therapy” OR “sing” OR “rhythm*” OR “tempo*” OR “Music Therapy”
4	Strategy 1 AND 2 AND 3
**Using SAGE Premier database**	
1	TI = “dementia” OR “cognitive impairment*” OR “cognitive dysfunctions” OR “neurocognitive disorder, mild” OR “cognitive decline” OR “mental deterioration”
2	AB = “dementia” OR “cognitive impairment*” OR “cognitive dysfunctions” OR “neurocognitive disorder, mild” OR “cognitive decline” OR “mental deterioration”
3	TI = “exercise” OR “train” OR “training” OR “physical activity” OR “physical activities” OR “strength” OR “endurance” OR “resistance” OR “stability” OR “walk*” OR “tai chi” OR “yoga” OR “motor control” OR “core control” “stretch*” OR “run” OR “muscle energy technique” OR “pilates*” OR “hydrotherapy” “water sports” OR “kinesitherapy”
4	TI = “Music” OR “music therapy” OR “sing” OR “rhythm*” OR “tempo*” OR “Music Therapy”
5	AB = “Music” OR “music therapy” OR “sing” OR “rhythm*” OR “tempo*” OR “Music Therapy”
6	Strategy 1 AND 2 AND 3 AND 4 AND 5
**Using EBSCO database**	
1	TI = “dementia” OR “cognitive impairment*” OR “cognitive dysfunctions” OR “neurocognitive disorder, mild” OR “cognitive decline” OR “mental deterioration”
2	AB = “dementia” OR “cognitive impairment*” OR “cognitive dysfunctions” OR “neurocognitive disorder, mild” OR “cognitive decline” OR “mental deterioration”
3	TI = “exercise” OR “train” OR “training” OR “physical activity” OR “physical activities” OR “strength” OR “endurance” OR “resistance” OR “stability” OR “walk*” OR “tai chi” OR “yoga” OR “motor control” OR “core control” “stretch*” OR “run” OR “muscle energy technique” OR “pilates*” OR “hydrotherapy” “water sports” OR “kinesitherapy”
4	TI = “Music” OR “music therapy” OR “sing” OR “rhythm*” OR “tempo*” OR “Music Therapy”
5	AB = “Music” OR “music therapy” OR “sing” OR “rhythm*” OR “tempo*” OR “Music Therapy”
6	Strategy 1 AND 2 AND 3 AND 4 AND 5
**Using Cochrane Library database**	
1	TI = “dementia” OR “cognitive impairment*” OR “cognitive dysfunctions” OR “neurocognitive disorder, mild” OR “cognitive decline” OR “mental deterioration”
2	AB = “dementia” OR “cognitive impairment*” OR “cognitive dysfunctions” OR “neurocognitive disorder, mild” OR “cognitive decline” OR “mental deterioration”
3	TI = “exercise” OR “train” OR “training” OR “physical activity” OR “physical activities” OR “strength” OR “endurance” OR “resistance” OR “stability” OR “walk*” OR “tai chi” OR “yoga” OR “motor control” OR “core control” “stretch*” OR “run” OR “muscle energy technique” OR “pilates*” OR “hydrotherapy” “water sports” OR “kinesitherapy”
4	TI = “Music” OR “music therapy” OR “sing” OR “rhythm*” OR “tempo*” OR “Music Therapy”
5	AB = “Music” OR “music therapy” OR “sing” OR “rhythm*” OR “tempo*” OR “Music Therapy”
6	Strategy 1 AND 2 AND 3 AND 4 AND 5

TI, Title; AB, Abstract; and TS, Topic (Title, Abstract, Author Keywords, Keywords Plus).

### Inclusion criteria

The inclusion criteria include: (1) patients diagnosed with dementia; (2) AD patients receiving combined physical activity and music intervention; (3) availability during the intervention and testing phases; (4) randomized controlled trials (RCTs), crossover study, case series/report, and single-subject design study; and (5) publications indexed in databases from January 2002 to March 2022 provided they are written in English.

### Exclusion criteria

**FIGURE 1:**
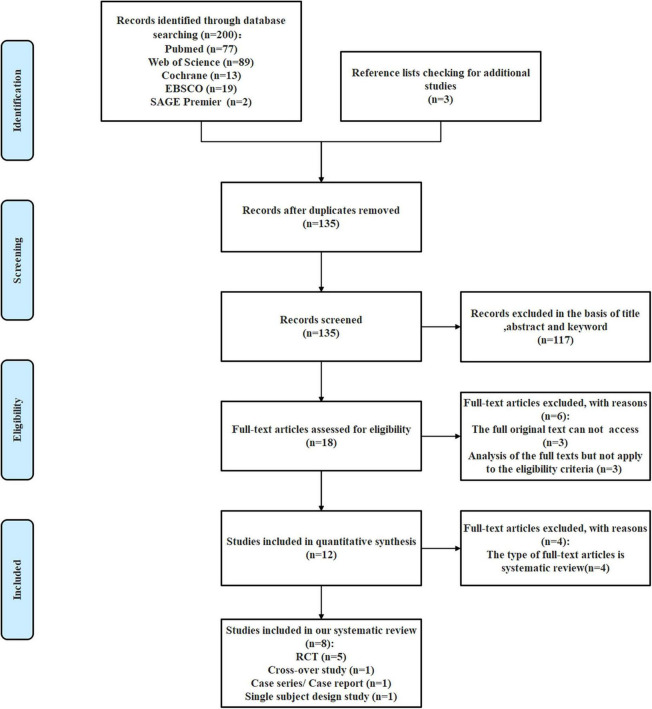
Flow chart.

The exclusion criteria include: (1) publications that do not have access to at least the abstracts; (2) documents that are not published in the form of a peer-reviewed article, such as dissertations, theses, conferences, editorial letters, and commentaries; (3) duplicate items; (4) participants with other diseases; (5) combined physical activity and music interventions that are not part of the treatment for patients with AD; and (6) the absence of combined physical activity and music intervention-related outcome measures. Figure 1 presents the flow chart of the search for bibliographic references of this study.

### Data extraction

All the potential studies were reviewed by two researchers independently. Abstracts and titles were reviewed and scored. If the abstracts and titles satisfied the criteria, the full texts would be examined to determine which research met the inclusion or exclusion criteria. If a disagreement ensued, a team discussion would be made by researchers to approach a final decision. In this processes, researchers extracted various details, including the information of authors and publication year, subject features, and intervention from studies. The outcomes of studies were also extracted.

### Quality assessment

The study quality was assessed by Grading of Recommendations Assessment, Development and Evaluation system, it was developed by Guyatt et al. (2011). In general, this system is for systematic review and health technology assessments, as well as guidelines (Guyatt et al., 2008). This appraisal tool consists of 15 items focused on study design, experimental control, study participants, methodology, and outcomes. The first item, addressing study design, is worth five points. Each additional item is worth one point, for a maximum score of 20. Both researchers ranked articles simultaneously, and disagreements were resolved to reach a consensus.

## Results

### Studies included and excluded

Our team of researchers identified two hundred studies from screening potential studies. After screening all studies. 68 duplicate records were removed. A total of 135 records were screened based on the title, abstract, and keywords; and 19 full-text articles were eligible. Finally, eight studies were included (see Figure 1 flow chart). Out of the eight studies, six examined the effect of combined physical activity and music treatment to AD patients, two studies explored the effect of cognitive stimulation (CS) by strengthening exercise and music therapy.

### Study designs

Five studies applied RCT design (Van de Winckel et al., 2004; Satoh et al., 2017; Chen and Pei, 2018; Pitkänen et al., 2019; Higuti et al., 2021), one study applied crossover design (Johnson et al., 2012), one used a case report design (Langhammer et al., 2019), and one applied a single-subject design (Mathews et al., 2001). The four RCTs included the treatment and control arms, and one RCT had more than two arms (three-armed RCT).

### Quality assessment tool applied

The quality assessment consisted of 15 items (see Table 2), the whole assessment tool worth 20 points. The items involved these categories, such as study design, experimental control, participants, methodology, and outcomes. In terms of this assessment tool, studies were marked as high, medium, and low quality. Articles were ranked simultaneously by researchers.

**TABLE 2:** Quality evaluation of references.

References	Higuti et al., 2021	Langhammer et al., 2019	Johnson et al., 2012	Satoh et al., 2017	Pitkänen et al., 2019	Van de Winckel et al., 2004	Mathews et al., 2001	Chen and Pei, 2018
*Study design criteria*	5	1	2	5	5	5	3	5
5-Randomized controlled trial								
4-Cohort study								
3-Single subject design study								
2-Crossover study								
1-Case series/Case report								
*Additional criteria (1 point each)*								
Clear experimental controls used	1	0	1	1	1	1	0	1
Prospective study completed	0	0	1	0	0	0	0	0
Blinding of assessors used	1	0	1	0	0	1	0	0
Clear description of subjects/group	1	1	1	1	1	1	1	1
Balanced baselines between groups or stable across single subject	0	0	0	0	0	0	0	0
Target behaviors observable and measurable	1	1	1	1	1	1	1	1
Clear description of intervention methods	1	1	1	1	1	1	1	1
Attrition rate explained or minimal (<20%)	1	1	1	0	1	1	0	1
Clear description of observable or measurable outcomes	1	1	1	1	1	1	1	1
Statistical analysis described or conducted appropriately	1	1	1	1	1	1	0	1
Appropriate reliability methods described or used	0	0	0	0	0	0	0	0
Appropriate validity methods described or used	0	0	0	0	0	0	0	0
Clear conclusions drawn from results	1	1	1	1	1	1	0	1
Clear description of follow-up and maintenance outcomes	0	0	0	0	0	1	0	0
*Total score*	14	8	12	12	13	15	7	13

Studies were then ranked as high (14–20 points), medium (7–13 points), and low quality (1–6 points).

The quality assessment is presented in Table 2. No low-quality study was detected. We obtained two studies that have high-quality, and six that are medium-quality. All studies have a clear description of subjects/groups, observable and measurable clear target behaviors, clear descriptions of intervention methods, and clear description of observable or measurable outcomes. Five studies were reported as RCTs, and one each of single-subject design study, crossover study, and case report study. The eight studies reported appropriate statistical analysis and clear conclusions drawn from the results.

### Combined physical activity and music intervention

Nowadays, music intervention has a similar purpose when compared to physical activity in patients with AD, but music intervention may use totally different techniques to achieve these goals. Music intervention utilizes methods such as rhythmic auditory stimulation (RAS), therapeutic instrumental music performance, and musical neglect training to patients’ movement (Wittwer et al., 2019; Braun Janzen et al., 2021). Studies about combined physical activity and music intervention are very limited, but it has proven important for patients with AD. For example, physical activity accompanied with RAS produced significant improvements in strength, functional activity promotion of synchronized movements.(Minino et al., 2021). In this current study, among these searched articles, music interventions always came with physical activities or movements, such as sitting and standing balance activities, or stretching, strengthening, and breathing exercises (Table 3). Also, Several studies utilized the intervention consisting of singing and playing instruments using a fast tempo and well-pronounced beat (Johnson et al., 2012; Chen and Pei, 2018; Ptomey et al., 2018; Langhammer et al., 2019). In addition, other music interventions consisted of music training for the upper and lower extremities while clapping to music (Satoh et al., 2017). Moreover, physical activities included walking on the ward or outside of it. Singing or listening to known songs was also among the interventions.

**TABLE 3:** Physical activity combined music for individuals with AD-current research summary table.

*N* (% completing study) and drop outs	Study design	Intervention group	Control group	Intervention conducted by	The length and frequency of the intervention of the study	Outcomes	Significant findings
Higuti et al., 2021 17 (94% completed) 1 dropped out (6%)	RCT	Group 1: the Training without Music Group performed a set of light exercises lasting 25–30 min per session focused mainly on maintaining/improving global mobility. Group 2:the Training with Music Group was initially submitted to cognitive stimuli with music.	No intervention	Physiotherapy students, researcher.	Once a week for 12 weeks; lasting 25–30 min per session; 6 of 12 sessions	Assess functional capacity: The BI and PPS Assess cognition: MMSE, 24 Severe MMSE, and VFT	No improvement or worsening in cognitive or functional aspects was found after 12 weeks of training in either group.
Langhammer et al., 2019 16 (100% completed) 0 dropped out (0)	Case series/Case report	A combination of physical activity (activities or movements such as sitting and standing balance activities, stretching, strengthening exercises, breathing exercises, and different sport activities) with balls music (activities or movements such as singing, playing musical instruments, moving to the music, or simply listening to enhance relaxation), and walking.	No intervention	Physiotherapist, music therapist	Once per week for 8 weeks; lasting 45 min per session	Brøset Violence Checklist. NPI-Q The primary caretaker (nurse assistant) documented compliance to the program by recording weekly activities in a logbook.	Implementation of a systematic combination of music and a physical activity program was feasible in a group of individuals with severe dementia, and helped reduce anxiety, restlessness, irritability, and aggression.
Johnson et al., 2012 12 (100% completed) 0 dropped out (0)	Crossover study	The intervention consisted of music during an exercise program. The musical selection included seven songs with a medium to fast tempo and a well-pronounced beat. An exercise program was developed to simulate common movements found in typical exercise. Exercises were choreographed with the beat of the music for the intervention condition.	The control condition consisted of the exercise program described above without music.	Care givers	Once per week for 8 weeks; lasting 30 min per session (including five 2-min breaks)	A chart was used to track participation. Each exercise session was divided into intervals of 30 s. Within these time intervals, reviewers examined each resident and assigned a mark of one if the resident participated in the correct exercises for the whole interval and a 0 if the participant stopped participating at any point during the interval.	The use of music may be considered a supplemental intervention utilized to increase participation in exercise programs for older adults with dementia. Increasing participation in exercise programs can increase strength, decrease risk of falls, and improve quality of life.
Satoh et al., 2017 62 (72% completed) 23 dropped out (28%)	RCT	The program consisted of muscle training for the upper and lower extremities, hand clapping to music, breath and voice training, and singing.	No intervention	Musicians, nursing-care facility staff (nurses, certified care workers, or psychiatric social workers)	Once per week for 6 months; lasting 45 min per session	Neuropsychological batteries. FIM, VSRAD.	Exercise with music produced greater positive effects on cognitive function and ADLs in patients with mild to moderate dementia than CS, excluding memory. Optimal interventions for dementia will likely be achieved by combining Exercise with music and CS.
Pitkänen et al., 2019 175 (50%completed) 173 drop out	RCT	The physical exercise interventions included balance, flexibility and strength training either when seated or standing, relaxation and exercise with a restorator. Music was the part of the exercise sessions, included singing or listening to familiar songs.	Medication and multidisciplinary treatment	Music therapist, physiotherapist, the chief physician and nursing director	Six times a week; lasting 45 min per time	NPI, MMSE, BI, ADCS-ADL	Physical exercise may have some positive effects for both neuropsychiatric symptoms and the level of functioning in some patients with dementia while no positive effects regarding music interventions were found.
Van de Winckel et al., 2004 25 (100% completed) 0 drop out	RCT	Patients attended a group- based exercise programme. The therapist used specific one-step verbal instructions, combined with continuous visual demonstration. The exercises focused on upper and lower body strengthening, as well as balance, trunk movements and flexibility training.	the therapist had a daily one-to-one conversation with each of participants separately. No music was played and patients were not asked to perform any movements.	Physical therapist	Daily per session for 3 months; lasting 30 min per session	MMSE, ADS 6, BOP scale	The present study suggests a beneficial effect of cognition using a music-based exercise programme in a group of patients with moderate to severe dementia.
Mathews et al., 2001 21 (100% completed) 0 drop out	Single subject design study	Use of rhythmic music during exercise activities. The sequence of exercises included: shoulder roll; hand flex and hand pronate/supinate; knees together/apart; arms across chest; knee extensions; bicep curls; toe taps and heel lifts; bucket lifts; rowing; marching; arm adduction/abduction and internal/external rotation; head rotation and ear to shoulder; ankle circles; and arm extensions.	No control group	Physical therapist	Once per week for 25 weeks; lasting 22 min per session	The observer used a 30-s partial interval recording system to score individual resident engagement.	Results showed increased levels of participation during the experimental condition observations where rhythmic music accompanied the exercise activities. The music intervention was most successful on those generally most willing to participate in social activities
Chen and Pei, 2018 28 (93.33% completed) 2 drop out	RCT	The MDTT protocol included a musical task and a walking task. The musical task comprised two types of activities: singing and playing simple percussive musical instruments. In the walking task, the participants either walked forward or stepped sideways. The participants were instructed to perform all eight combinations progressively within a session.	Non-musical cognitive and walking activities.	Music therapist	Once per week for 8 weeks; lasting 60 min per session	TMT part A, Army Individual Test Battery, dual-task performance in gait analysis included the forward digit recall dual-task condition, backward digit recall dual-task condition, and single-task (control) condition, TUG test, FES-I, CMAI-C	MDTT intervention demands a high level of cognitive processing, enhances attention control, falls efficacy, and helps alleviate agitation in patients with mild-to-moderate dementia.

RCT, randomized controlled trial; BI, barthel index; PPS, palliative performance scale; MMSE, the mini mental state examination; VFT, verbal fluency test; NPI-Q, neuropsychiatric inventory–questionnaire; FIM, the functional independence measure; VSRAD, voxel-based specific regional analysis system for Alzheimer’s disease; ADL, activities of daily living?; CS, cognitive stimulation ?; NPI, neuropsychiatric inventory; ADCS-ADL, Alzheimer’s disease cooperative study–activities of daily living; ADS 6, amsterdam dementia screening test 6; BOP scale, stockton geriatric rating scale; MDTT, musical dual-task training; TMT part, trail making test part A; TUG, Timed Up and Go; FES-I, falls efficacy scale international; and CMAI-C, chinese community-version cohen-mansfield agitation inventory scale.

### Music therapy with cognitive stimulation

Cognitive stimulation has various methods such as: using portable game consoles and drills involving easy calculations, mazes, and mistake-searching in pictures (Satoh et al., 2017). Satoh et al. (2017) performed a CS in which the subjects utilized the software named “Yawaraka-atama-juku” (which means “flexible thinking club”). Higuti et al. (2021) applied CS using seven extraordinary songs relevant to their age, evoking positive personal memories. After the CS test, participants were guided to perform the same type of exercises.

### Dosage of interventions

In these eight studies, the dosage of each intervention ranged from 1 to 25 weeks. Each intervention session was lasting 22 to 45 min. These studies did not mention the unsupervised adherence rates, thus, we presumed that per week adherence of the same duration as guided by investigators (Gilat et al., 2021).

### Assessment tool used in the eight studies

Barthel index (Satoh et al., 2017; Pitkänen et al., 2019; Higuti et al., 2021) and functional independence measure were used to assess the daily functional score. Mental health was assessed by the mini-mental state examination (MMSE; Van de Winckel et al., 2004; Pitkänen et al., 2019; Higuti et al., 2021). Psychological, functional, and health status were used to evaluate the cognition situation. Pitkänen et al. (2019) used a scale tool called Alzheimer’s disease cooperative study-activities of daily living.

Langhammer et al. (2019) and Pitkänen et al. (2019) performed neuropsychiatric inventory to assess neuropsychiatric condition. Other behavioral and functional assessments used included dual-task performance in gait analysis, such as the forward and backward recall the number dual-task condition, and single-task (control) condition, timed up-and-go test (TUG; Chen and Pei, 2018); 30-s partial interval recording system for scoring individual resident engagement (Mathews et al., 2001); and Stockton Geriatric Rating Scale (Van de Winckel et al., 2004).

Other studies have used different assessment tools to evaluate the neuromuscular function of patients with AD, such as switching the mind attention dual-task, paired associative learning, and decreasing the reaction time (Ptomey et al., 2018).

### Study population

Table 2 summarizes the information on each of the 8 studies we explored in this systemic review. The participants from each group in the last 8 studies ranged from 6 to 175, with a median of 29 patients in each group. The intervention groups had 51% male and 41% female (49%), whereas the control groups had 41% male and 59% female. The sex of the participants in the two studies was not reported (Van de Winckel et al., 2004; Satoh et al., 2017). The average age for all the groups was 81.6 (3.8) years, with the intervention groups demonstrating and average of 80.4 (3.7) years, and the placebo groups an average of 79.8 (3.1) years. The range was between 53 and 94 years. The number of years since clinical diagnosis was recorded in four studies, and the Clinical Dementia Rating has been reported in the study (Higuti et al., 2021). The group averages on the MMSE were described as an accurate and effective cognitive screening test in three studies (Van de Winckel et al., 2004; Pitkänen et al., 2019; Higuti et al., 2021).

### Results obtained

Previous systematic reviews have examined the effect of music or physical exercise in isolation. This review was the first to conclude that the combination of these interventions improved cognitive and physical function, and quality of life of AD populations. Langhammer et al. (2019) found that combining physical activity with music therapy is effective for reducing anxiety, restlessness, irritability, and aggression in a sample of people with severe dementia. Johnson et al. (2012) concluded that music may be used as a supplemental element to increase participation of physical exercise programs for patients with AD. Increasing exercise involvement may enhance strength, and reduce the risk of falling. Mathews et al. (2001) showed that music accompanying exercise activities increased the levels of participation during experimental condition observations. Those who were most eager to participate in activities benefited the most from music-based intervention. Furthermore, Chen and Pei (2018) noted that in patients with mild to moderate dementia, music combined with physical exercise intervention needs a high level of cognitive processing. Therefore, this treatment enhances their attention and fall efficacy, and can reduce their agitation. Satoh et al. (2017) further stated that combined music and physical exercises are more effective than a single intervention for prevention of AD.

No significant association regarding music-based physical activity in patients with AD were found in the other two studies. Pitkänen et al. (2019) noted that exercise has effects on neuropsychiatric symptoms and the function among some AD patients, but it has no positive effects relative to music based interventions. Higuti et al. (2021) noted no enhancement or decline with both cognitive or functional characteristics among AD patients after 12-week intervention.

## Discussion

This systematic review was performed to assess the influence of combined physical and music intervention as a strategy to improve function and quality of life for patients with AD. Nowadays, there are various studies available regrading physical therapy and music therapy treatment to patients independently (Guo et al., 2020), but current studies using a collaboration of these two disciplines is limited. Applying music to stimulate a sense of rhythm will assist to stimulate certain brain areas and improve the synchronized physical exercise (Matthews, 2015; Song and Ryu, 2016; Zhang et al., 2017). This current review indicates that irrespective of the treatment modalities or combination chosen, music-based physical activities can act as a non-pharmacological treatment beneficial to patients with AD. Supporting combined physical and music intervention will play a key role in helping clinical guidelines for both physical therapists and music therapists.

### Effect of combined physical activity and music intervention to patients with Alzheimer’s disease

Most of the reviewed studies identified the effect of combined physical activity and music treatment on cognitive and physical functions of patients with AD (Mathews et al., 2001; Van de Winckel et al., 2004; Satoh et al., 2017; Chen and Pei, 2018; Ptomey et al., 2018; Langhammer et al., 2019; Giuli et al., 2020). This phenomenon could be due to the fact that combined physical activity and music intervention helps patients with mild to moderate dementia reduce anxiety, restlessness, irritability, and aggression. Increased participation among physical exercise programs can increase strength for patients (Johnson et al., 2012), reduce risks of falls, and promote quality of life (Langhammer et al., 2019). The music-based physical exercises effect on the functional and cognitive characteristics of older persons with mild to advanced dementia were explored in two studies. Depending on the form or stage of dementia, exercise before or after music intervention, such as listening to music can assist in slowing the development of dementia, and can be used in conjunction with existing cognitive behavioral and pharmacological therapies (Moreno-Morales et al., 2020).

No modern technologies had been applied to support combined physical activity and music intervention among the previous studies that we analyzed. A number of technologies for patients with AD have emerged over the past 20 years, and other modern technologies, such as cellphones or wearable fitness trackers, could be examined to assist patients with dementia engage in physical activities while listening to music (van der Wardt et al., 2017). In addition, patients with dementia may use modern technology, and smartphones, apps, and other similar technologies may soon become the standard, as they are expected to be used by the majority of people on a regular basis (Rosenberg et al., 2012; Rosenberg and Nygård, 2014). Consequently, older patients with dementia may be familiar with such equipment.

The effectiveness of adherence techniques is expected to differ between patients with AD. No strong findings have been found about the usefulness of an adherence support program, and existing evidence is weak. More evidence is needed to determine what works best for whom and under what conditions. Thus, traditional systematic reviews are unlikely to provide solutions, necessitating the adoption of a realism epistemology.

### Clinical guidance

**FIGURE 2:**
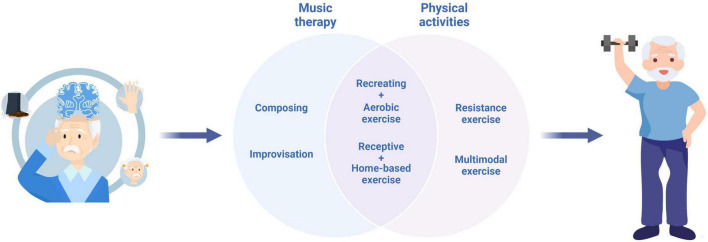
Combined physical activity and music interventions for patients with AD.

Various individual factors influence the responses of patients with AD to combined physical activity and music intervention. These key factors include, but are not limited to age, gender, cognitive function, familiarity with, and preference for the music-based physical activity types. The potential success of combining music and physical activities provides professionals, therapists and researchers a new treatment option to study and apply. The review showed that these treatments could help modify the progression of decline seen among people with dementia, as well as improve daily abilities for AD patients (Higuti et al., 2021). Per what is reviewed above, we would like to offer the following clinical guidelines for the use of combined physical activity and music intervention for practices. Figure 2 shows the major combined physical activity and music interventions for patients with AD.

• To improve the physical and cognitive functions of AD patients, the environment needs to be adjusted to promote music-based physical activities. One major modification in implementing health care is to update the physical environment to a more home-like environment, and suggest physical exercises that fulfill the needs of patients with AD (Richards et al., 2015). At the same time, playing the patients’ preferred music combined with exercise has the adjunct effect on cognition in patients AD (Li et al., 2015; van der Wardt et al., 2017).
• Various types of combined physical activity and music interventions should be explored in collaboration with people with AD, as well as their caregivers. Moreover, some of these tactics can be utilized in public health programs to encourage this population to participate in exercise classes or physical activity programs (van der Wardt et al., 2017).
• With the purpose of conducting interventions in this population, highly trained professionals who can comprehend and acculturate the signals and requirements of AD patients are vital.

### Research direction

In view of relatively weak evidence, the effectiveness of adherence support strategies varies among patients with AD. A larger sample of RCTs is urgently needed to evaluate the types of combined physical activity and music intervention, the population for whom it is used, the circumstances in which it is used, and explanation for why it is used. Few studies have analyzed the mechanism of music-based physical activities for patients with AD and how they improve cognition, mood and behavior of patients with AD. Furthermore, the development of new combined physical activity and music interventions should be explored in collaboration with people with AD, as well as their caregivers. Such interventions will assist in guiding health experts, such as physical therapists, so that a rehabilitation approach to dementia care can be implemented, thereby improving the quality of life of patients with AD.

## Study limitations

Although it has many strengths, this review also has some weaknesses. First, the three most truthful world databases used in this study gathered a large amount of scientific publications. However, future revisions should also search through more databases. Second, the term of “music therapy” or “music based intervention” can have several meanings. In this study, we have attempted to unify the term based on the definition of the World Federation of Music Therapy. However, some publications may apply different implications of this term, which can modify the results found within the bibliographic search. Third, the treatment standards of music combined with different exercise types, such as music with aerobic exercise, music with resistance exercise, music with combined aerobic and resistance training, music with home-based exercise, and music with multimodal exercise, are lacking. Fourth, only English-language studies were considered in this paper. Thus, several studies in other languages may have been overlooked.

## Conclusion

This systemic review attempted to state the feasibility and efficacy of combined physical activity and music intervention for patients with AD. The results of this systemic review support that combined physical activity and music interventions have been proved to be preferred, acceptable, and effective for patients with mild to moderate AD. In addition, combined physical activity and music interventions that combine strength, balance, flexibility, and endurance are the most common combinations that produce major improvements in the health of patients with AD.

## Data availability statement

The original contributions presented in this study are included in the article/supplementary material, further inquiries can be directed to the corresponding author.

## Author contributions

All authors contributed toward data analysis, revising the manuscript and agreed to be accountable for all aspects of the work. KL and H-YH designed and performed the study and drafted and revised the manuscript. H-YH, HZ, LJ, and RL analyzed the results and were involved in the research design and execution. CC revised the manuscript.
